# Structural Particularities of Gall Neoformations Induced by *Monarthropalpus flavus* in the Leaves of *Buxus sempervirens*

**DOI:** 10.3390/plants14030453

**Published:** 2025-02-04

**Authors:** Irina Neta Gostin, Irinel Eugen Popescu, Cristian Felix Blidar

**Affiliations:** 1 Faculty of Biology, Alexandru Ioan Cuza University of Iași, Bdul Carol I, No. 11, 700506 Iasi, Romania; 2 Department of Biology, Faculty of Informatics and Sciences, University of Oradea, Street Universităţii No. 1, 410087 Oradea, Romania; cblidar@gmail.com

**Keywords:** anatomy, Cecidomyiidae, histochemistry, gall inducer, leaf, microscopy, neo-formed tissue, SEM

## Abstract

The boxwood leafminer *Monarthropalpus flavus* (Diptera, Cecidomyiidae) has historically been considered a leafminer, but some researchers suggested it induced galls on *Buxus* species leaves. The larvae of *M. flavus* create small blister-like galls on *Buxus sempervirens* leaves, causing tissue hypertrophy and hyperplasia. Histological examination reveals that *M. flavus* larvae cause the formation of small blister galls, which involve tissue reorganization in the mesophyll. Unlike typical leafminers, which only disrupt existing tissues, *M. flavus* induces the appearance of a neo-formed tissue, near the larval chamber. This tissue, originating primarily from spongy parenchyma cells, significantly increases as the leaf thickens. Various histochemical analyses show that the new tissue contains starch, lipids, terpenes, and proteins, providing evidence of reprogramming in the plant’s metabolism. The study concludes that *M. flavus* induces rudimentary galls, not simply mines, due to the formation of new tissue, whose cells have cytological characteristics distinct from those found in non-galled leaves. However, despite some gall-like features, it does not create new vascular elements, distinguishing it from more complex galls formed by other gall-inducing species.

## 1. Introduction

*Buxus sempervirens* L., commonly known as boxwood, is a member of the Buxaceae family, which consists of six genera and approximately 100 species (POWO, 2024) [1]. The genus *Buxus* has two centers of diversification, one in the Latin American region (sect. Tricera) and the other in Eurasia (sect. Buxus) [2].

It is an evergreen shrub that can develop into a small tree when allowed to grow naturally [3,4]. Valued for its dense foliage, it is often shaped into hedges, topiaries, and other ornamental forms. Beyond its aesthetic value, *Buxus sempervirens* has been used in traditional medicine for its potential medicinal properties, including antioxidant, cytotoxic, anti-inflammatory, antidiabetic, antibacterial, and antifungal effects. However, caution is advised due to its toxicity [4]. Ecologically, it provides shelter for small wildlife and contributes to soil stabilization.

The interactions between plants and insects are diverse, ranging from commensal to mutualistic or antagonistic. Insects can pollinate plants, aiding in reproduction, spreading seeds, or offering protection, while others feed on, parasitize, or harm plants to ensure their own reproduction [5,6,7]. These relationships have evolved over 400 million years since the Devonian period [5]. In response, plants have developed strategies to attract, repel, or defend against insects [8], while insects continuously evolve mechanisms to bypass these defenses [9].

Herbivory, after pollination, is another key interaction, influencing plant dynamics, ecosystems, and the evolution of both plants and insects. Endophagous insects are categorized based on their interaction with host plants, including gall inducers, mining insects, borers, and inquilines, which live in structures created by other species [10,11].

The gall inducers and mining insects (specialized endophagous) utilize the plant body and any newly formed tissues as a source of food and shelter from potentially harmful exogenous factors. Unlike mining insects, gall inducers produce significant structural modifications to the plant organs they inhabit, leading to the formation of new anatomical structures—galls—which represent deviations from the normal morphogenesis of the respective plant species. The gall inducer has the ability to reprogram the plant’s development to create novel tissues that provide nutritional resources and sometimes protection for the insect, often at the expense of the plant’s growth and reproduction [12]. Most authors agree that mining insects do not generate structural modifications in the host plant; their feeding pattern is limited to already existing tissues, without the formation of newly created tissues with a nutritional role [13,14].

*Monarthropalpus flavus* (Schrank) (Diptera, Cecidomyiidae) (syn. *Monarthropalpus buxi* Labolt.), commonly known as the boxwood leafminer, is considered one of the major pests of *Buxus sempervirens*, causing significant damage to the plant [15]; it was traditionally classified as a member of the group of mining insects [16,17,18] rather than as a gall midge. However, some researchers consider this species belonging to the group of organisms that induce galls on various species of *Buxus* [19]. There is some variability in how this issue is approached, as the species’ feeding behavior is not universally interpreted in the same way by different authors. For example, Hamilton [20] describes the changes induced in the leaves by *M. buxi* as “gall-like swelling” and “gall-like growth caused by the larvae”. Similarly, Mitchell and colab. [21] refer to *M. flavus* as the “*Buxus* leaf-mining gall-midge”, while d’Eustachio and Raupp [22] use both the terms “mined leaf” and “gall formation” due to the presence of specialized tissue, visible even macroscopically.

Larvae of *M. flavus* cause the formation of small blister galls on both sides of the leaves of *Buxus sempervirens* [23]. Eiseman and Blyth [24] also note the occurrence of leaf blisters on *Buxus*. Meyer and Maresquelle [25] describe the rudimentary gall induced by *M. buxi* on the leaves of *B. sempervirens*, classifying the neoformation that appeared in the leaf structure as galls.

This study aims to investigate the anatomical alterations caused by the boxwood leafminer on *Buxus sempervirens* leaves, in order to clarify whether the insect behaves as a leafminer or a gall inducer. By examining the histological changes induced by the mining activity, this research contributes to a deeper understanding of the consequences of leaf modifications for both the plant and the insect, as well as the broader implications for plant–insect coevolution.

## 2. Results

### 2.1. Gall Inducer—Monarthropalpus flavus

**Figure 1:**
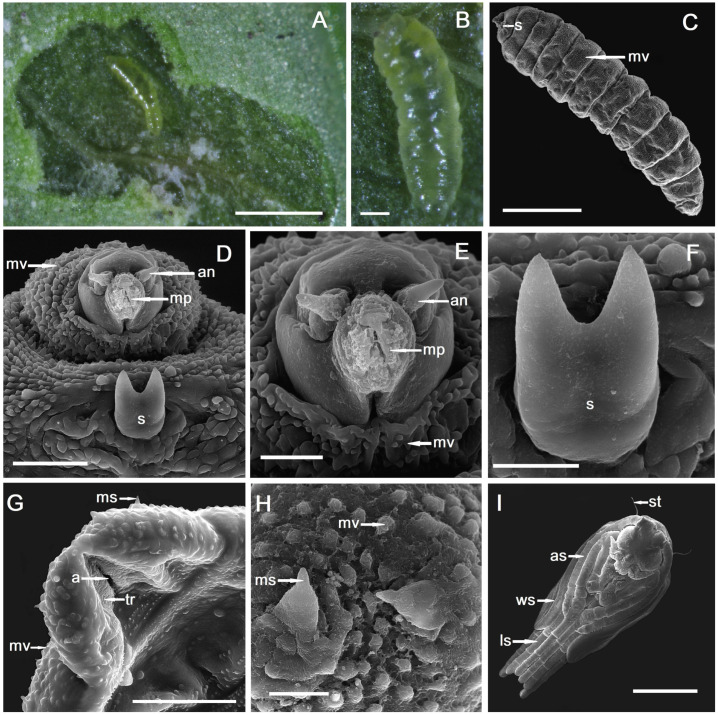
*Monarthropalpus flavus* larvae and SEM micrographs of larvae: (**A**,**B**) second instar larva, (**C**) third instar larva with sternal spatula (s), microverrucae (mv), (**D**) third instar larva with sternal spatula (s), microverrucae (mv), head with antenna (an), mouthparts (mp), (**E**) third instar larva with microverrucae (mv), head with antenna (an), mouthparts (mp), (**F**) sternal spatula (s) on third instar larva, (**G**) terminal part of the third instar larva with anus (a), transversal ridges (tr), microverrucae (mv), microsensilla (ms), (**H**) terminal part of the third instar larva with microsensilla (ms) and microverrucae (mv), (**I**) pupa with setae (st), antennal sheath (as), wing sheath (ws), leg sheath (ls). (**A**,**C**,**I**) Scale bar = 500 µm, (**B**) scale bar = 100 µm, (**D**,**G**) scale bar = 50 µm, (**E**,**F**,**H**) scale bar = 20 µm.

The larvae of *Monarthropalpus flavus* develop as endophagous inside the mesophyll of leaves of *Buxus* and are gregarious, group feeders, having the same larval stage, coming from the same mass of eggs. The body of larvae is elongate, cylindrical, and on the second instar larva with transparent cuticle and yellow colour, without a sternal spatula (Figure 1A,B).

The third instar larva is hemicephalic, asymmetrically fusiform (Figure 1C), with yellow–orange colour. The cuticle of the third instar larva is minutely verrucose (cuticular verrucae) with the verrucae making transversal lines helping in locomotion with the role of traction ridges (Figure 1C). There is a small sternal bilobed spatula on the ventral side of the prothorax (Figure 1D,F), used for perforating the plant tissues. On the terminal part of the third instar larva, there are some microsensilla and, around the anus, there are some shallow transversal ridges (Figure 1G,H). Pupa is exarate, with no visible apical spines but with two long setae (Figure 1I).

### 2.2. Non-Galled Leaf Anatomical Features

**Figure 2:**
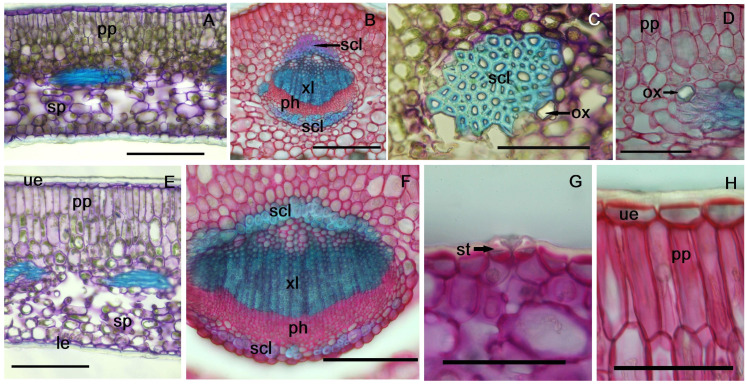
Cross-section through 1st year leaf (**A**–**D**) and 2nd year leaf (**E**–**H**): (**A**) lamina between veins, 1st year leaf, (**B**) detail of midrib, (**C**) detail of sclerenchyma sheath at the periphery of the leaf blade, (**D**) detail of palisade parenchyma (pp) and a prismatic crystal of calcium oxalate, (**E**) lamina between veins, 2nd year leaf, (**F**) detail of midrib, (**G**) stomata (st) in the lower epidermis, (**H**) detail of palisade parenchyma: le—lower epidermis, pp—palisade parenchyma, ph—phloem, scl—sclerenchyma, sp—spongy parenchyma, st—stomata, ue—upper epidermis, xl—xylem, ox—calcium oxalate crystals. (**A**,**B**,**E**,**F**) Scale bar = 100 µm, (**C**,**D**,**G**,**H**) scale bar = 50 µm.

In cross-section, the leaf has a bifacial structure (Figure 2A,B). Both epidermises are single-layered with flattened cells, and the external walls are thickened, especially in the lower epidermis (6.21–7.4 µm). Stomata are present only in the lower epidermis (hypostomatous leaf), positioned at the same level as the epidermal cells, with a distinct external ridge bordering the ostiole (Figure 2G).

**Figure 3:**
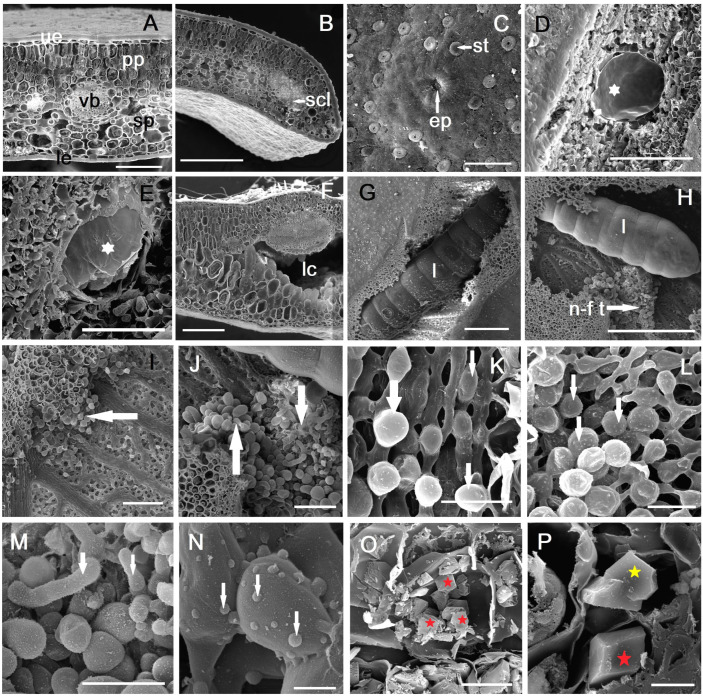
Scanning electron microscopy (SEM) images: (**A**,**B**) cross sections through a normal leaf, (**C**) egg insertion area in the lower epidermis, (**D**) empty larval chamber (in early stage—white star), (**E**) larval chamber with 1st instar larva inside (early stage—white star), (**F**) cross section through leaf with mature gall (near the midrib), (**G**,**H**) gall with 3rd instar larva inside, (**I**) neo-formed tissue in proximity to spongy parenchyma (white arrow), (**J**) neo-formed tissue near actively feeding larva (white arrow), (**K**–**N**) stages of development of neo-formed tissue in gall (white arrows), (**O**,**P**) calcium oxalate crystals in the vicinity of gall (red star—simple calcium oxalate crystals, yellow star—compound calcium oxalate crystals): ep—entry point, l—larva, le—lower epidermis, lc—larval chamber, n-f t—neo-formed tissue, pp—palisade parenchyma, scl – sclerenchyma, sp—spongy parenchyma, st—stomata, ue—upper epidermis, vb—vascular bundle. (**H**) Scale bar = 1 mm, (**G**) scale bar = 500 µm, (**B**,**D**,**F**,**I**,**J**) scale bar = 200 µm, (**A**,**C**,**E**,**M**) scale bar = 100 µm, (**K**,**L**) scale bar = 50 µm, (**O**) scale bar = 20 µm, (**N**,**P**) scale bar = 10 µm.

The mesophyll consists of palisade parenchyma beneath the upper epidermis and spongy parenchyma beneath the lower epidermis (Figure 3A). In one-year-old leaves, the palisade parenchyma has 3–4 layers of cells, with the first two being elongated and the others nearly isodiametric (Figure 2A,D, Table S1). In two-year-old leaves, the palisade parenchyma has two layers of elongated cells (18–23 µm) and two layers of shorter cells near the center (Figure 2E,H).

The midrib protrudes more on the abaxial side; this presents a collateral vascular bundle, with secondary xylem, formed by numerous small vessels. On either side of the bundles, at both the xylem and phloem poles, two sheaths of sclerenchyma fibers are observed; these consist of 2–3 layers of cells with strongly thickened and lignified walls (Figure 2B,F). At the edge of the lamina, two sheaths of sclerenchyma fibers, not associated with the conductive tissue, are also observed (Figure 2C and Figure 3B). In the mesophyll, prismatic or compound calcium oxalate crystals are observed, located predominantly near the veins, some associated with vascular bundles (Figure 2C,D). Additionally, in the second year of the leaves’ life, after the cold season, a separation between the two assimilatory tissues (palisade and spongy) is observed, more pronounced in the midrib area and sometimes absent towards the edges of the leaf (Figure 2E,F).

### 2.3. Anatomy of Galled Leaves

**Figure 4:**
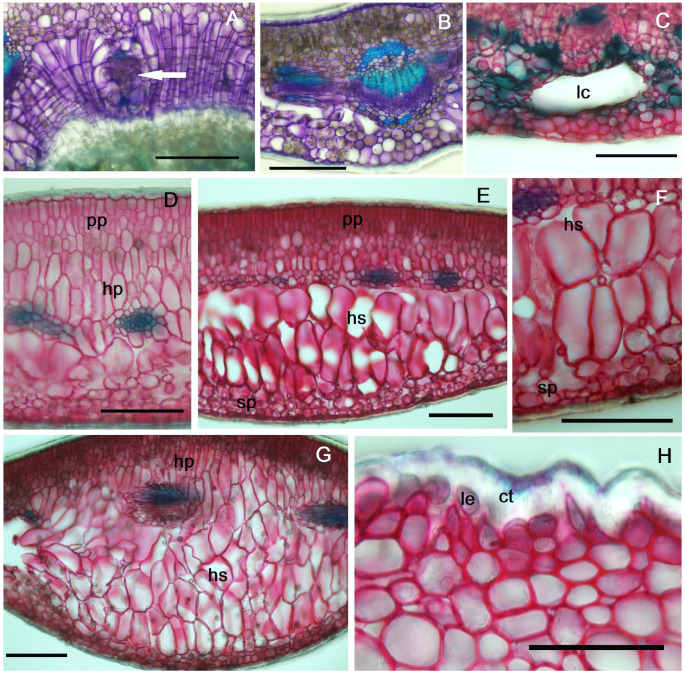
Cross-section through gall in first year leaf: (**A**,**B**) cross-section through leaves sampled in May: (**A**) initiation zone of galls (white arrow), (**B**) area near the gall, in the vicinity of the midrib, (**C**–**G**) cross-sections through leaves sampled in August; (**C**) larval chamber, (**D**–**G**) details from the vicinity of the larval chamber showing hyperplasia and hypertrophy of cells originating from the assimilatory tissue, (**H**) detail from the lower epidermis with modified cells: ct—cuticle, hp—hypertrophied tissue towards palisade parenchyma, hs—hypertrophied tissue towards spongy parenchyma (HTHS), lc—larval chamber, le—lower epidermis, pp—palisade parenchyma, sp—spongy parenchyma. (**A**–**G**) Scale bar = 100 µm, (**H**) scale bar = 50 µm.

The point of insertion of the insect egg into the leaf is visible on the lower epidermis (Figure 3C). At the point of entry, a scar tissue forms on the outside of the leaf, and in the mesophyll, a reaction of hyperplasia and hypertrophy of the assimilatory cells is observed (Figure 4A). The larval chamber initially has small dimensions (up to 150–200 µm), and a mucilaginous layer is observed on the periphery (Figure 3D,E).

Once the larva starts feeding, the two assimilatory tissues (palisade and spongy) in the mesophyll begin to separate (Figure 3F and Figure 4B). Near the initial larval chamber a slight lignification occurs, as a response by the host plant to the gall-inducer attack (Figure 4C). The interaction between the larva and the host plant leaf induces hyperplasia, followed by hypertrophy of the cells derived from the assimilatory tissue (Figure 3D–G). The neo-formed tissue cells are larger towards the spongy parenchyma than those in the proximity of the palisade parenchyma (Figure 4D–F). As the larva grows and moves into the thickness of the mesophyll, larval sites (blisters) grow and merge; usually, in a leaf, several larvae can be observed in a large, common chamber (Figure 3G,H). Neo-formed tissue is observed in this cavity, in the vicinity of the larvae, though it is not uniformly distributed (Figure 3I,J); it usually appears in clusters, in the vicinity of the veins where nutritional resources are more abundant (although this is not always the case).

In the area of the fully formed gall, in August, the thickness of the leaf blade increases significantly, nearly three times (Table S1); by successive divisions, new cell layers appear (5–8). The growth occurs exclusively based on the development of the neo-formed tissue, because the height of the cells in the palisade parenchyma and spongy parenchyma does not change significantly (Table S1). At the level of the cells in the lower epidermis (Figure 4H), near the midrib, a modification is observed consisting of a change in their shape and an increase in the thickness of the cuticle, largely due to the increasing pressure exerted by the rapid growth in the thickness of the leaf lamina.

Scanning electron microscopy (SEM) observations clearly reveal the origin of neo-formed tissue through a process of “budding” from spongy parenchyma cells that undergo divisions in the area of contact with the larval chamber. Subsequently, these cells increase in volume, becoming spherical or oval, and have a different shape from the spongy parenchyma cells from which they originate (Figure 3K–M).

**Figure 5:**
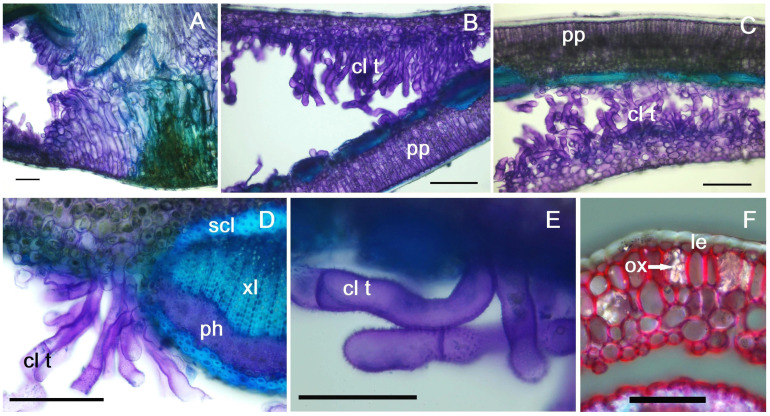
Cross-section through gall in second year leaf: (**A**) cross-section through mature gall, (**B**,**C**) callus-like tissue filling the areas of the leaf where the larva has fed, (**D**) callus-like cells developed in the vicinity of the midrib, (**E**) detail of the cells of the callus-like tissue, (**F**) calcium oxalate crystals observed under polarized light: cl t—callus-like tissue, le—lower epidermis, ox—calcium oxalate crystals, ph—phloem, pp—palisade parenchyma, scl—sclerenchyma, xl—xylem, (**A**–**D**) scale bar = 100 µm, (**E**,**F**) scale bar = 50 µm.

After the formation of the galls and the movement of the larva to another feeding place, a callus-like tissue develops from the hypertrophied cells, with elongated, tubular cells (Figure 3J,M), particularly visible in the second-year leaf (which survives even after the adult hatches) (Figure 5A,B). These cells are particularly observed near the spongy parenchyma, with the tubular cells sometimes arranged in rows of 2–4, stacked uniseriately (Figure 5B,C). On the external side of the cell walls (which are of a primary nature, cellulose–pectic) verrucosities can be observed, more pronounced in cells located at the apical area of the tube (Figure 3N and Figure 5E). When the tubular cells grow near the vascular bundles, no interactions are observed, and the vascular architecture remains unchanged (Figure 5D). In the areas affected by galls, especially in their late stages of development, prismatic calcium oxalate crystals of various sizes can be observed (Figure 3O,P and Figure 5F): they may be located one or two in the cell, if they are larger, or more abundant (over 10–15) if they are smaller in size.

### 2.4. Histochemical Profiles of the Galls

**Figure 6:**
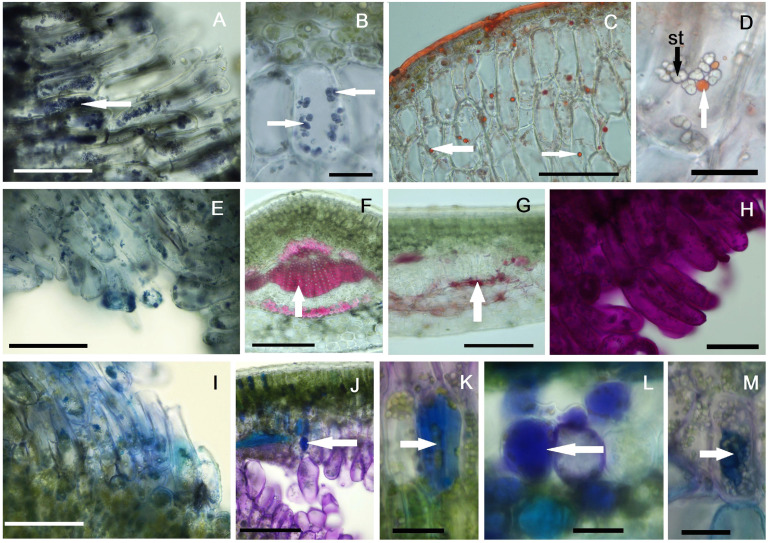
Histochemical profiles of the galls: (**A**,**B**) starch stained in violet with Lugol’s reagent (white arrows), (**C**,**D**) lipids stained in red with Sudan Red (white arrows), (**E**) terpenes stained in dark blue with NADI reagent, (**F**,**G**) lignin stained in purple red with phloroglucinol (white arrows), (**H**) polysaccharides stained in red with PAS reagent, (**I**) proteins stained in blue with Coomassie brilliant blue, (**J**–**M**) polyphenols stained in blue with toluidine blue and Na_2_CO_3_ (white arrows); st—starch, (**A**,**C**,**E**,**F**,**G**,**I**,**J**) scale bar = 100 µm, (**B**,**D**,**H**,**K**–**M**) scale bar = 25 µm.

Histochemical analyses of the plant material, represented by mature galls on one-year-old leaves, showed positive results for most reactions, except for the Erlich reagent test for IAA identification, which was negative in all the investigated galls. Starch was identified with Lugol’s solution in large quantities in the neo-formed tissue cells (Figure 6A). Starch granules are compound and present only in the modified tissue, not in the adjacent spongy parenchyma cells (Figure 6B). Red Sudan III identified lipid in large quantities in the cuticle, as expected, as well as in the form of droplets in the neo-formed tissue cells (Figure 6C,D). These lipids are absent or very rare in the assimilatory tissues located distal to the larval chamber.

The NADI reagent test for terpenes recorded positive results in the neo-formed tissue cells (Figure 6E), while the reaction in the assimilatory tissue was only weakly positive. The identification of lignin using phloroglucin and hydrochloric acid produced positive results in the xylem vessels and periphloemic sclerenchyma fibers (Figure 6F); also, lignin spots were detected in the vicinity of the larval chamber, during the early stages of gall development (Figure 6G). Later, in the hypertrophied cells of the neo-formed tissue, the reaction was consistently negative.

The PAS reaction for the identification of polysaccharides was positive both in the assimilatory mesophyll and in the cells of the neo-formed tissue (Figure 6H), without having an express specificity for it. Proteins, identified histochemically with Coomassie brilliant blue, were observed in large quantities in the neo-formed tissue (Figure 6I), as well as in the vascular bundles, phloem and wood parenchyma cells. Polyphenolic compounds were predominantly identified in the cells of the normal assimilatory tissue (mainly in palisade parenchyma), but were also present in some cells of the neo-formed tissue (Figure 6J–M).

## 3. Discussion

Many authors have considered, over time, that the larva of this species digs mines in *Buxus* leaves, feeding with their assimilatory tissue [17,18,26,27]. Even the widely accepted popular name—“boxwood leafminer”—reflects this feeding behavior of the insect larva.

In general, the specialized literature makes a clear distinction between the two categories of endophagous insects: gall inducers and leafminers [10,28]. However, some opinions suggest that even leafminer insects have the ability to reprogram the plant organism [12].

Galls represent the peak of the interaction between phytophagous insects and host plants. These insects do not only obtain food from the host plant, but their manipulation of plant development by gall inducers leads to complex tissue reorganization, which can sometimes result in the formation of new plant organs [29]. Due to the diversity of galls, both structurally and in terms of the insect groups that cause them, providing an exact definition of the term is difficult [30]. Redfern [31] defines a gall as a growth or swelling caused by hypertrophy (enlargement) and/or hyperplasia (multiplication) of plant cells, induced by an organism, which provides nutrients/food and shelter for that organism”. Fernandes and collaborators [32] attempt a broader definition of galls to encompass their various degrees of differentiation. They consider galls to be “new multicellular organs generated by coordinated cell division and expansion”. Therefore, the defining condition for an organism to be considered a gall inducer is its ability to cause changes in the metabolism and morphogenesis of the host plant, creating an environment conducive to its development. In contrast, leaf-mining insects are a diverse group of phytophagous organisms that feed on plants by creating tunnels, or “mines”, within plant tissues [33]. Mines typically disrupt the leaf’s mesophyll tissue without causing swelling or excessive growth [30]. The mines, like the galls, are formed by the larvae of the insects, which are endophagous, meaning they live and feed internally within the plant organs. In this way, the plant plays a dual role for the insect—it provides a food source and shelter for development, offering protection from external predators and environmental stressors [33]. Leafminers are considered an intermediate primitive form of endophagy [12], while gall inducers possess more evolved forms of exploitation of the host plant to obtain nutritional and protective advantages [34].

Histo-anatomical investigations on the galls produced by *M. flavus* in the leaves of *B. sempervirens* are scarce. Del Bene and collaborators [35] describe the formation of “blister galls”, focusing on the life cycle of the gall midge rather than on the peculiarities of the neo-formed tissue and the quantitative changes that occur in the leaf lamina following gall development. The hypertrophy of the assimilatory cells and their degeneration with the hatching of the adult is mentioned; however, the development of callus-like tissue, which begins with the active feeding process of the larva and continues in the leaf from the second year, is not mentioned. Meyer and Maresquelle [25] describe a callus that develops in the larval cavity and originates from the spongy parenchyma. However, contrary to our observations, the authors state that starch is absent from the cells lining the larval chamber, but is abundant in the palisade parenchyma.

The detailed analysis of the modifications induced in the leaf of *B. sempervirens* by *M. flavus* leads to the conclusion that it produces a rudimentary gall or blister gall, rather than being a simple leafminer. The main argument is the presence of neo-formed tissue, which originates from both the spongy parenchyma cells (predominantly) and the palisade parenchyma cells. Hypertrophied cells of the neo-formed tissue, intensely vacuolated and containing various categories of nutrients, are perforated by the larva with the sternal bilobed spatula and the contents are sucked into the mouth [20].

The presence of polyphenols in the cells of leaves affected by galls is reported in numerous species in the literature. Their role is not consistently defined, with some authors suggesting that polyphenols serve as a defense mechanism of the host plant against the parasite [36,37], while others propose that the synthesis of polyphenols is induced by the parasite in the host plant to protect it from potential enemies [38,39,40]. In the case of *M. flavus*, the presence of polyphenols in cells, especially in the palisade parenchyma, suggests a host-plant defense response, as the larva consumes very little of the palisade parenchyma during its development. Although they are not frequently found in gall tissues, terpenoids were identified in neo-formed tissue from *B. sempervirens* leaves. Similar results were obtained through histochemical testing with NADI reagent in galls produced by cecidomyiids in lateral buds of *Marcetia taxifolia* (Melastomataceae) [41].

The near-intact preservation of the palisade parenchyma, which ensures the continuity of photosynthetic activity at the site of gall development, has been described in the case of galls induced by the cecidomyiid *Schismatodiplosis lantanae* on the leaves of *Lantana camara* [42]. Although in the case of galls produced by *M. flavus,* the neo-formed tissue also originates from palisade parenchyma cells, the predominant source is spongy parenchyma, which is also the most affected by gall induction. Thus, the tissue with the lowest performance in terms of capacity of photosynthesis is “sacrificed” to satisfy the nutritional needs of the larva, while the functional maintenance of the palisade parenchyma ensures the survival of the leaf and, implicitly, the maintenance of the shelter necessary for the insect.

Regarding starch, our investigations showed the presence of numerous amyloplasts in the cells of the neo-formed tissue, including those near the larval chamber. These cells lack chloroplasts. In contrast, few starch granules were observed in the cells of the palisade parenchyma, and they were absent in the cells of the spongy parenchyma. A similar starch distribution was observed by Guimarães and collaborators [43] in the gall tissue produced by *Clusiamyia nitida* (Cecidomyiidae), in the leaves of *Clusia lanceolata*, as well as in Cecidomyiidae galls of *Aspidosperma spruceanum* [44]. However, starch in the newly formed tissue cannot be used by the larva or for gall formation, so carbohydrate conversion is required. Oliveira and collaborators [44] found glucose-6-phosphatase activity in Cecidomyiidae galls, aiding sucrose formation. However, this is not the common pattern found in galls induced by this family of insects; in most cases, starch is absent from the tissues in the vicinity of the larval chambers [45].

Lipids, identified as nutritional substances by histochemical methods in galls, are particularly specific in the case of lepidopterans [46]. Our investigations on the gall induced by *M. flavus* highlighted lipid droplets in neo-formed tissue cells, although these are in small quantities. Histochemical analysis also revealed the presence of proteins, with an increasing gradient from the larval chamber towards the two epidermises of the leaf. A higher concentration was observed in the vascular bundles, but this is also visible in non-galled leaves. Protein buildup around the larval chamber is linked to respiratory stress and increased plant cell metabolism [47]; proteins have also been reported as a nutritional source for cecidomyiids [45].

The phenomenon of lignification was not strongly evidenced by our histochemical research—only a few cells with lignified walls were identified near the larval chamber during the early stages of development. Although many cecidomyiid galls develop specialized sclerenchymatous tissue surrounding the larval chamber in a protective role [48], in the case of the gall induced by *M. flavus*, this process seems to primarily serve as a defense against external aggression. However, the gall inducer later inhibits lignin production, as lignification is only observed in the xylem and sclerenchyma of the mature gall, which are also present in non-galled leaves.

The presence of calcium oxalate crystals, especially in the vicinity of the larval chambers, has also been reported in other galls induced by cecidomyiids in different plant species: *Lopesia* sp. on leaves of *Mimosa gemmulata* [49], *Clinodiplosis profusa* on leaves of *Eugenia uniflora* [50]. The role of calcium oxalate in gall tissues, and in plant tissues in general, has not yet been fully clarified [51]. It is assumed that, in young galls, it would be a sign of intense metabolic activity [52], and subsequently protects both the plant and the gall inducer from herbivorous natural enemies [49]. In the case of galls induced by *M. flavus*, the peripheral positioning of calcium oxalate crystals (usually under the epidermis) and not in the vicinity of the larval chamber supports the hypothesis of protection from other potential herbivores, rather than the gall inducer.

Gall development is traditionally divided into four stages: induction, growth and development, maturation, and dehiscence [29]. However, in the case of the genus *Buxus*, which has evergreen leaves in the temperate climate where it grows (with all the adaptive structural features that allow this), the senescence phase (observable in summer in second-year leaves) is different. Typically, the end of the larval feeding activity and the transition to the pupal stage is accompanied by the degeneration of the hypertrophied tissue in the gall, which served as a food source [53]. One of the structural changes in the normal *Buxus* leaf, occurring with passage through the cold season, namely, the separation of the palisade parenchyma from the spongy parenchyma, at least in the central area of the leaf [54], is also induced by the development of the common larval chamber following infestation with *M. flavus*. However, the leaf is adapted to survive with this modification, and although it occurs early, it does not lead to the death of the leaf. The neo-formed tissue does not degenerate after the insect leaves the larval chamber; on the contrary, its cells grow significantly, becoming tubular, overlapping, and partially filling the empty space of the larval chamber. Maintaining the functional and structural integrity of the palisade parenchyma allows some leaves to survive the following year, despite the damage caused by the infestation.

Although most gall midges (Cecidomyiidae) produce complex galls, with specialized nutritive tissue and a sclerenchymatous layer that separates the larval chamber from the rest of the parasitized organ [55], *M. flavus* induces rudimentary galls in the leaves of *B. sempervirens*, which, however, involve more significant changes in its morphogenesis than the simple mines created by leafminers. In addition, the larval morphology shows particularities related to the strictly phytophagous feeding mode: the tegument is without long sensilla trichodea, as in *Lasioptera rubi* (Schrank) [56], which help disseminate the fungus in the tissue of the gall, the larva of *M. flavus* being just phytophagous and not phytomycetophagous as is the larva of *L. rubi* [56].

The investigated structure shows similarities to a classic gall, including hypertrophy, hyperplasia, alterations in tissue cellular structure, and the appearance of newly formed tissue. However, there are key differences: it does not affect the vascular tissue, and no new vascular elements are observed in the newly formed tissues. Furthermore, unlike galls induced by cecidomyiids, there are no meristematic formations in the neo-formed tissue that generate new vascular elements [39,43,56].

A deeper understanding of the interaction between the larva and the host plant can help identify more effective methods to combat this pest. To date, control methods have primarily targeted adults [22] rather than larvae [15], using conventional insecticides that are toxic to both humans and the environment. Given the increasing importance of biopesticides in combating various pest species [57], future studies on the species’ sensitivity to these substances are necessary.

## 4. Materials and Methods

### 4.1. Plant Material

Mature one-year-old (n = 50) and two-year-old (n = 50) leaves of *Buxus sempervirens* were collected in August 2023 from the city of Iași (Romania), Podul de Piatră area (47°09′24.4″ N 27°34′27.1″ E). In addition, young leaves (n = 10) were harvested in May, from the same location, to observe the initial stages of gall development.

### 4.2. Light Microscopy

The galls were dissected with razor blades and micro dissecting needles under a Euromex stereomicroscope (Euromex Microscopen BV, Arnhem, The Netherlands) with a maximum magnification of 180×. Images were obtained using a Canon 60D digital camera attached to a ZEISS SteREO Discovery.V20 stereomicroscope (ZEISS Microscopy, Oberkochen, Germany).

### 4.3. Histo-Anatomical Investigations

The plant material (galled and non-galled leaves) was fixed in 70% ethyl alcohol, and then manually sectioned using a razor blade and a microtome. The sections were stained with Iodine Green and Ruthenium Red [58,59] or with Toluidine blue [60]; microphotographs were taken based on the most relevant aspects using an Olympus BX41 research microscope (Olympus Corporation, Tokyo, Japan) equipped with an Olympus E-330 camera.

Measurements of anatomical parameters for one-year-old leaves, including palisade heightfirst layer (PH1), palisade height—second layer (PH2), spongy tissue cell height (SH), spongy parenchyma thickness (SPT), palisade parenchyma thickness (PPT), upper epidermis cell height (UEH), upper epidermis cuticle height (UECH), lower epidermis cell height (LEH), lower epidermis cuticle height (LECH), mesophyll thickness (MT), hypertrophied tissue cell height—towards palisade parenchyma (HTHP), and hypertrophied tissue cell height—towards spongy parenchyma (HTHS), were carried out using IC Measure Imaging Source software (version 2.0.0.286; The Imaging Source, LLC, Charlotte, NC, USA).

The means of the anatomical parameters listed above (with the exception of the last two values HTHP and HTHS, which are not found in normal leaves) were compared using Student’s *t*-test. The differences were considered significant when *p* < 0.05.

### 4.4. Histochemical Investigations

Freshly harvested plant material (mature galls from one-year-old leaves) was manually sectioned and subsequently stained with various reagents to highlight specific classes of compounds in the affected leaves. Unstained sections served as a control. The reagents used were Lugol’s solution for starch detection [61], Sudan Red III for lipid substances [62], NADI reagent for terpenes [63], acidified phloroglucinol for lignin [61], PAS reagent for polysaccharide identification [64] (McManus, 1948), Coomassie brilliant blue for proteins [65], toluidine blue and Na_2_CO_3_ for the presence of polyphenols [66,67], and Ehrlich reagent for IAA (indoleacetic acid) [68].

### 4.5. Scanning Electron Microscopy Investigations

The samples (leaf fragments with and without galls, larvae and pupae) were fixed in 70% ethyl alcohol. Plant samples were sectioned with a razor blade to highlight the affected areas of the leaves, as well as to expose the normal structure of the unaffected leaves. Dehydration was performed in an increasing series of alcohols (80%, 90%, and 100%, twice), followed by two acetone baths (p.a.). Subsequently, they were dried at the critical point of CO_2_ (using an EMS 850 Critical Point Dryer, Hatfield, PA, USA). The samples were mounted on stubs with carbon conductive tape, and sputter coated with a thin layer of gold (30 nm) using a sputter coater (EMS 550X Sputter Coater, Hatfield, PA, USA). Observations were made with the Tescan Vega II SBH scanning electron microscope (TESCAN, Brno, Czech Republic), from the Electron Microscopy Laboratory, Faculty of Biology, “Alexandru Ioan Cuza” University of Iaşi.

## Data Availability

The original contributions presented in the study are included in the article; further inquiries can be directed to the corresponding authors.

## Supplementary Materials

The following supporting information can be downloaded at: https://www.mdpi.com/article/10.3390/plants14030453/s1, Table S1. Anatomical parameters of normal and galled leaves (first year) induced by *Monarthropalpus flavus* in *Buxus sempervirens*.
