# Supplementary material for: Structural Particularities of Gall Neoformations Induced by Monarthropalpus flavus in the Leaves of Buxus sempervirens
Source: Plants (Basel). 2025 Feb 4;14(3):453. doi: 10.3390/plants14030453 (PMC11821084; doi:10.3390/plants14030453)
Supplement: Supplementary file 1 [file plants-14-00453-s001.zip › plants-3426892-supplementary.pdf]

Table S1. Anatomical parameters of normal and galled leaves (first year) induced by *Monarthropalpus flavus* in *Buxus sempervirens* (n=15): palisade height - first layer (PH1), palisade height - second layer (PH2), spongy tissue cell height (SH), spongy parenchyma thickness (SPT), palisade parenchyma thickness (PPT), upper epidermis cell height (UEH), upper epidermis cuticle height (UECH), lower epidermis cell height (LEH), lower epidermis cuticle height (LECH), mesophyll thickness (MT), hypertrophied tissue cell height - towards palisade parenchyma (HTHP), hypertrophied tissue cell height - towards spongy parenchyma (HTHS). The values presented in the table represent the means ± standard deviation.

Leaf type	Normal leaf (µm)	Galled leaf (µm)
PH1	23.62±2.21[a]	23.25±2.41[a]
PH2	18.57±3.59[a]	20.21±4.13[a]
SH	12.49±1.52[a]	14.03±3.49[a]
SPT	87.81±5.75[a]	41.85±6.36[b]
PPT	57.64±1.84[a]	49.01±7.97[b]
UEH	5.34±0.8[a]	5.71±0.89[a]
UECH	7.4±0.52[a]	6.96±0.79[a]
LEH	5.84±0.99[a]	5.96±0.78[a]
LECH	6.21±1.2[a]	5.68±0.87[a]
MT	171.02±8.07[a]	315.08±46.39[b]
HTHP	-	47.18±5.08
HTHS	-	56.87±11.26

[1] Different lowercase letters denote significant differences ($P < 0.05$) among the anatomical parameters from normal and galled leaves.
